# Novel *SOX17* frameshift mutations in endometrial cancer are functionally distinct from recurrent missense mutations

**DOI:** 10.18632/oncotarget.20213

**Published:** 2017-08-12

**Authors:** Christopher J. Walker, Matthew J. O'Hern, Vanida A. Serna, Takeshi Kurita, Mario A. Miranda, Caroline E. Sapp, David G. Mutch, David E. Cohn, Paul J. Goodfellow

**Affiliations:** ^1^ James Comprehensive Cancer Center and the Department of Obstetrics and Gynecology, The Ohio State University, Columbus, OH 43210, USA; ^2^ James Comprehensive Cancer Center and the Department of Cancer Biology and Genetics, The Ohio State University, Columbus, OH 43210, USA; ^3^ Siteman Cancer Center and the Department of Obstetrics and Gynecology, Washington University School of Medicine, St. Louis, MO 63110, USA

**Keywords:** transcription factor, endometrial cancer, SOX gene, SRY-box, SOX17

## Abstract

Extensive genomic profiling for endometrioid endometrial carcinoma (EEC) has pointed to genes and pathways important in uterine development as critical mediators of endometrial tumorigenesis. SOX17 is a developmental transcription factor necessary for proper endoderm formation that has been implicated as a tumor suppressor and shown to modulate WNT signaling. *SOX17* mutation analysis in 539 primary EECs revealed frequent missense and frameshift mutations with an overall 11.5% mutation rate. More than half the mutations identified were frameshifts (32 of 62), and the hotspot missense changes, p.Ala96Gly and p.Ser403Ile, were seen in 14 tumors. None of the cases with a mutation had a second *SOX17* mutation or evidence of allelic loss. Immunofluorescence microscopy performed on primary samples showed that there were no changes in SOX17 protein expression associated with mutation. Low/absent SOX17 staining was significantly associated with advanced stage, high tumor grade and reduced recurrence-free survival. Functional assessment of the two hotspot missense mutations and three representative frameshift mutations showed that SOX17-A96G and SOX17-S403I have transcriptional activities similar to SOX17 wild-type (WT), whereas none of the frameshift mutant proteins showed transcriptional activity. Forced expression of SOX17-WT, -A96G or -S403I in EC cell lines moderately increased β-catenin mediated transcription, which contrasts with previous data showing SOX17 is an inhibitor of TCF/β-catenin signaling. The proliferation of EC cell lines was expectedly reduced by transfection with SOX17-WT, and further reduced by SOX17-A96G and SOX17-S403I. These data implicate *SOX17* mutation as a selected event in EEC, with clear differences between the missense and frameshift mutations.

## INTRODUCTION

Endometrial cancer (EC) is the most common gynecologic malignancy and both the incidence and associated mortality of EC are increasing [1]. ECs are broadly classified into two groups: about 85% of ECs are endometrioid endometrial carcinomas (EECs) and are called type I, whereas type II ECs are non-endometrioid histology (mainly serous, mixed or clear cell) [2, 3]. The genomic landscape of EEC is well established and includes mutations in oncogenes and tumor suppressor genes involved in a variety of cancers, as well as genes that play distinct roles in the disease [4–8]. Type I tumors are frequently mutated in *PTEN*, *PIK3CA*, *ZFHX3, CTCF, MAX*, *PIK3R1*, *FBXW7* and *CTNNB1*, have few large somatic copy number alterations (SCNAs) [5], and frequently display microsatellite instability (MSI) [9, 10]. The most frequently mutated genes in type II tumors are *TP53, PIK3CA, PTEN, PIK3R1*, and *PPP2R1A*, and these tumors have many more SCNAs than type I ECs [5]. EC has one of the highest mutation rates among cancers [11], and thus many of the less frequently occurring mutations require biologic characterization to determine their importance in EC. The roles of these genes that are less frequently mutated in EEC, in particular those with known roles in uterine development, warrant further investigation.

SOX17 is a critical transcription factor that specifies endoderm lineages during development [12, 13]. It is essential for uterine adenogenesis during development and shows high expression in the adult uterus [14, 15]. Ectopic expression of SOX family members, and forced expression of a mutant form of SOX17 can induce de-differentiation of somatic tissues [16, 17]. Not surprisingly, SOX abnormalities have been associated with cancers [18]. *SOX17* transcription is repressed in various solid tumors through epigenetic and other mechanisms, and it has been implicated as a tumor suppressor in EC [19–23]. Sequencing efforts by The Cancer Genome Atlas (TCGA) identified somatic *SOX17* mutations in EECs, including two recurrent hotspot missense changes. Here we report that frameshift and stop mutations in *SOX17* are more common in EECs and functionally different than missense changes.

## RESULTS

### Both frameshift and missense mutations frequently occur in *SOX17* in EEC

**Figure 1:**
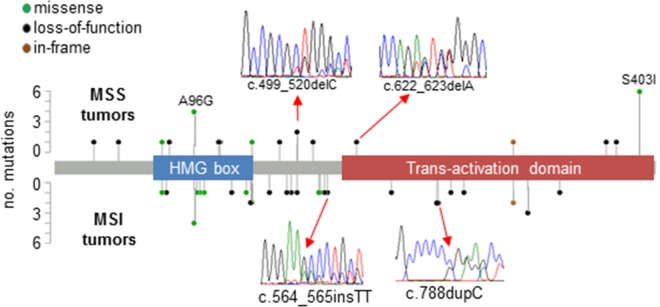
Pattern of mutations in *SOX17* in EECs Lollipop plots show somatic mutations in *SOX17* in 539 EEC samples stratified by DNA mismatch repair status (microsatellite instability positive [MSI] and microsatellite stable [MSS] tumors). Inserts show examples of Sanger sequencing traces from mutant tumors. Forty-two different mutations were observed in 62 tumors, including the hotspot missense changes p.Ala96Gly (observed in MSS and MSI tumors), and p.Ser403Ile (exclusively seen in MSI tumors). A complete description of mutations can be found in Supplementary Table 1.

*SOX17* was sequenced in 539 EECs with 42 different somatic mutations observed in 62 tumors (overall mutation rate = 11.5%, Supplementary Table 1). No tumor harbored more than one *SOX17* mutation, and there was no evidence for loss of heterozygosity (deletion of the wild-type allele) in the mutated tumors (data not shown). Frequent missense and loss-of-function (LOF) mutations were both observed (Figure 1). Thirty-two tumors harbored frameshifts, 25 tumors had missense mutations, 3 tumors had in-frame indels and 2 tumors had nonsense mutations (Supplementary Table 1). The two hotspot missense mutations previously identified by TCGA for EC [5] were evident: p.Ser403Ile was seen in eight tumors and p.Ala96Gly was seen in six tumors. Of the 12 different missense changes seen, 10 are predicted to have deleterious effects on protein function using Condel prediction software [24] (Supplementary Table 1).

The presence of a *SOX17* mutation was significantly associated with MSI, a hyper-mutated phenotype caused by defects in DNA mismatch repair present in ∼30% of EECs (Supplementary Table 2). Most of the frameshift mutations seen were not, however, strand-slippage mutations typically associated with MSI. Only four of the 26 different frameshifts occurred in repetitive DNA sequences (p.E264fs*101, p.P263fs*124, p.R273fs*114, and p.P328fs*59, all of which involved C repeats). The C repeat mutations were seen in MSI tumors only (Supplementary Table 1). Twenty-eight tumors in our series have *POLE* proofreading domain mutations that have been associated with an ultra-mutated phenotype (10 *POLE*-mutated tumors are MSI and 18 are MSS) [5, 25, 26]. Only one *POLE*-mutated tumor harbored a *SOX17* mutation and this mutation was likely not mutational noise secondary to the *POLE* defect since it was not a TCT>TAT or TCG>TTG transition [27]. Together these data are consistent with *SOX17* being under cancer specific mutational selection in this tumor type.

*SOX17* mutation status was significantly associated with tumor grade. Fifty percent of *SOX17*-mutant tumors were grade 2, compared to 33% of *SOX17* wild-type (WT) tumors (Supplementary Table 2). Mutation status was not associated with lymphovascular space invasion or Fédération Internationale de Gynécologie et d’Obstétrique (FIGO) stage, or with patient age, race and body-mass index (Supplementary Table 2). There was no association between *SOX17* mutation status and outcome (data not shown).

### Low SOX17 expression is associated with poor outcome

**Figure 2:**
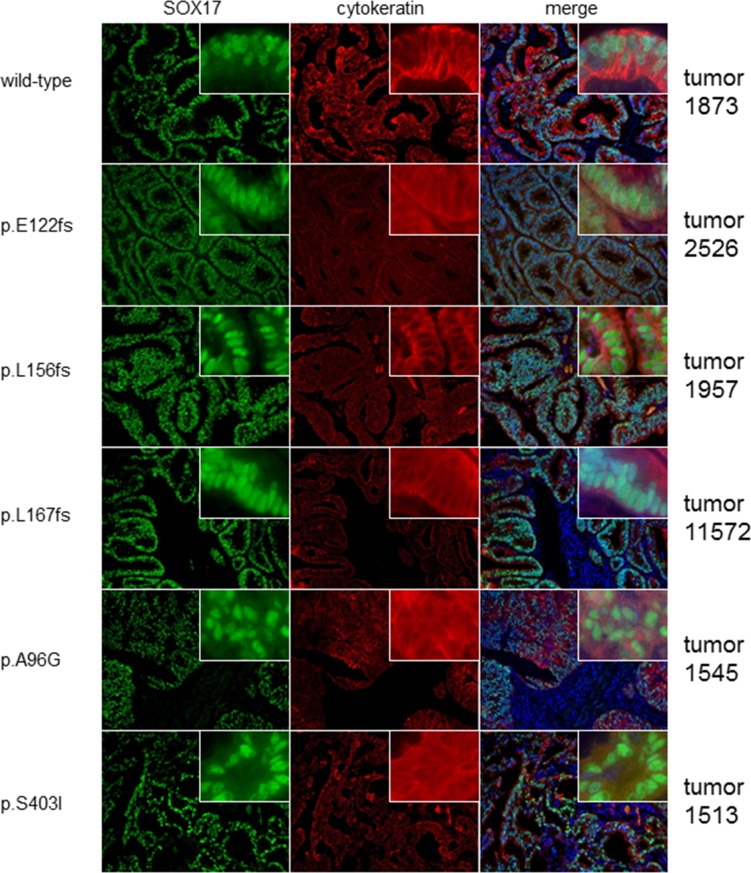
Mutated and wild-type tumors display similar SOX17 expression patterns Photomicrographs show SOX17 protein levels in one representative *SOX17* wild-type (WT) tumor and five tumors harboring different *SOX17* mutations. Tumors are counter-stained with cytokeratin to show epithelial tissue. SOX17 shows strong nuclear staining in the epithelial components of all tumors, regardless of mutation status.

To test the hypothesis that *SOX17* mutation is associated with changes in protein expression we used immunofluorescence microscopy to analyze expression in 51 primary EECs (37 wild-type and 14 with mutations). The majority (45 of 51) had detectable SOX17 protein. The staining pattern was as expected: SOX17 localized to cell nuclei, was prominent in the glandular epithelial cells and absent in the stromal compartment (Figure 2). Consistent with a previous report [23], advanced stage tumors were more likely to have low/absent SOX17 expression (Supplementary Table 3), and tumors with low/absent SOX17 staining had significantly reduced recurrence-free survival (RFS) (Supplementary Figure 1). There was also a significant association between high grade tumors (grades 2 and 3) with absent/low SOX17 expression (Supplementary Table 3). However, there was no difference in expression levels between the wild-type and mutant cases (Figure 2 and Supplementary Table 3). Because *SOX17* is a two-exon gene, the transcripts from frameshift mutations should escape nonsense-mediated decay, but we were unable to detect putative truncated protein products in most cases because the antibody we used for this staining recognizes an epitope that is C-terminal to most of the frameshift mutations investigated in this experiment (Asp177-Val414). Therefore, the comparable expression levels in tumors with wild-type *SOX17* and those with single N-terminal frameshifts are consistent with allelic compensation.

Because *SOX17* can be epigenetically silenced through promoter methylation we hypothesized that those tumors with absent SOX17 expression might have hypermethylated promoters [28–30]. Combined bisulfite restriction analysis (COBRA) of the 420bp *SOX17* promoter region was performed for five tumors with low/absent SOX17 expression and four with medium/high protein expression. There was very little methylation in this region present in these samples, with only one tumor, 1655T, showing any discernable methylation by COBRA (Supplementary Figure 2A). We confirmed sample 1655T had some methylated CpG sites by directly sequencing TOPO clones from amplified bisulfite converted DNA. We found that 3 of 11 of the PCR clones from tumor 1655T contained at least one intact restriction site, whereas the PCR clones from the COBRA-negative tumor 1484T did not contain any intact sites (Supplementary Figure 2B).

### *SOX17* frameshift and missense mutants have different transcriptional activities

**Figure 3:**
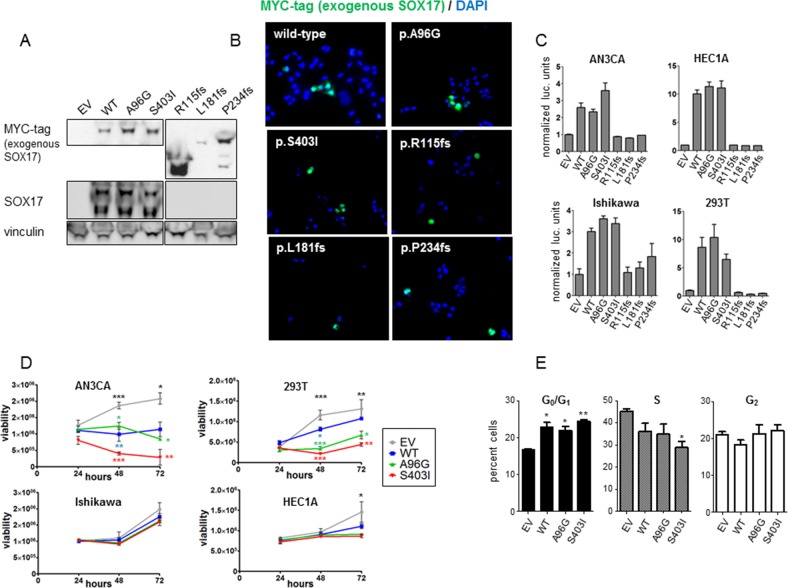
*In vitro* characterization of *SOX17* mutations **(A)** Western blot of 293T cells with forced expression of myc-tagged SOX17-WT or mutant protein. Exogenous SOX17 was detected using an anti-myc antibody and total SOX17 was detected using a SOX17 antibody. The SOX17 frameshift proteins have the expected myc-tagged SOX17 band sizes, but are not detectable with the anti-SOX17 antibody due to the mutations. Vinculin is loading control. Endogenous SOX17 is evident in all lysates with longer exposure (not shown). The endogenous lower mass truncated SOX17 isoform is also detected by the SOX17 antibody [51]. **(B)** AN3CA cells were transfected with myc-tagged SOX17-WT or the indicated SOX17 mutant constructs. Immunofluorescence microscopy shows none of the mutations alter SOX17 localization to the cell nucleus. **(C)** Transcriptional activity of wild-type and mutant SOX17 on a compressed SOX-OCT reporter. The ratio of firefly to renilla luciferase is shown, normalized to empty vector (EV). Error bars are standard deviation of biologic triplicates. **(D)** Effects on cell growth. Viability of the indicated cell lines transfected with wild type and mutant SOX17 was measured 24, 48, and 72 hours after transfection using Cell Titer-Glo assays. Error bars are standard deviation of technical duplicates. Experiments were performed in three biologic replicates and one representative trial is shown. Significance determined for each time point by one-way ANOVA (black asterisks) and Tukey's multiple comparison test (colored asterisks indicate comparison to empty vector) * *P* < 0.5; ** *P* < 0.01; *** *P* < 0.001. **(E)** AN3CA cells were transfected with EV, SOX17-WT, SOX17-A96G or SOX17-S403I, and the percentages of cells in different stages of the cell cycle was assessed using propidium-iodide staining and flow cytometry. Experiment performed in triplicate with 10,000 recorded events per condition per trial. Asterisks denote significant differences compared to EV determined by Tukey's multiple comparison test. * P<0.05; ** P< 0.01.

To investigate the effects of *SOX17* mutations on protein function, myc-tagged WT-SOX17 and representative SOX17 mutants (A96G, S403I, R115fs, L181fs, and P234fs) were expressed in 293T cells and three different EC cell lines. Western blot of 293T cells transiently transfected with the WT and mutant expression constructs showed expression of all five mutants, with lower mass bands (smaller protein) in the frameshift mutant lanes (Figure 3A). Immunofluorescence microscopy of transfected AN3CA cells proved that all mutant proteins were localized to the nucleus (Figure 3B), consistent with retention of the predicted nuclear localization sequence in all of the mutants. SOX17 transcriptional activity was assayed using a SOX17-activiated luciferase reporter that harbors two compressed SOX-OCT DNA binding sequences (5’ CATTGTATGCAAAT 3’) [16] (Supplementary Figure 3). Both the SOX17-A96G and SOX17-S403I mutants showed transcriptional activities similar to SOX17-WT in 293T cells and three different EC cell lines (AN3CA, HEC1A and Ishikawa), whereas the frameshift mutations did not show any activity above baseline (Figure 3C). To test if the frameshift mutant proteins were capable of inhibiting transcriptional activity, SOX17-WT was co-transfected with the mutant constructs. SOX17-driven transcription was not reduced by co-expression with the frameshift mutant proteins, indicating that these mutations were not acting as dominant negatives (Supplementary Figure 4).

It has been reported that forced expression of SOX17 reduces proliferation of cancer cell lines [21, 23, 31]. To test if the mutations modulated SOX17's effects on proliferation, SOX17-WT, SOX17-A96G and SOX17-S403I constructs were transfected into four different cell lines and the viability was measured daily (Figure 3D). To determine if the differences in proliferation were due to changes in cell death or growth rate, propdium-iodide cell cycle analysis was performed on AN3CA cells transfected with EV, SOX17-WT, SOX17-A96G and SOX17-S403I. The SOX17-WT and SOX17-mutant expressing cells had increased fractions of cells in the G_0_/G_1_ phase, pointing to changes in cell cycle accounting for the differences in proliferation (Figure 3E).

### *SOX17* missense mutations do not affect β-catenin expression or activity

**Figure 4:**
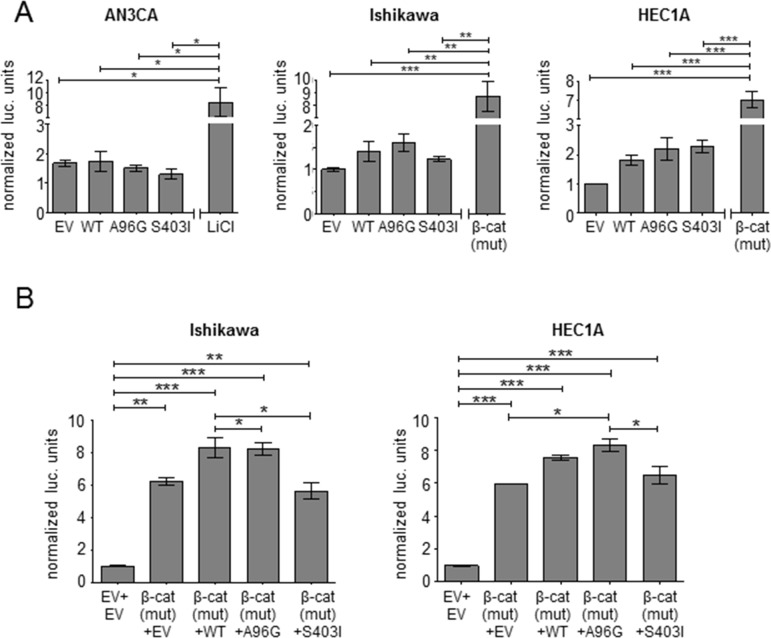
SOX17 does not repress TCF/β-catenin activity in EC **(A)** SOX17 effects in the context of basal TCF/β-catenin-mediated transcription. Transcriptional activity of the pBAR TCF/β-catenin activity reporter measured in the indicated endometrial cancer cell lines after transfection with empty vector (EV), SOX17-wild-type (WT) or the indicated SOX17 mutants. LiCl treatment or transfection of a quadruple mutant β-catenin (S33A, S37A, T41A, S45A) with enhanced stability were used as controls to activate the reporter. **(B)** SOX17 effects in the context of elevatedTCF/β-catenin-mediated transcription. Co-transfection of the indicated SOX17 construct (or EV) with quadruple mutant β-catenin and the luciferase plasmids was performed in the indicated cell lines. The first lane shows basal reporter activity without mutant β-catenin transfection. Firefly to renilla luciferase ratio is reported (normalized to EV). Significance determined by Tukey's multiple comparison test, * *P* < 0.5; ** *P* < 0.01; *** *P* < 0.001.

SOX17 can be induced by β-catenin and can also act as a WNT signaling antagonist in a variety of normal and cancerous tissues [32–34]. To explore the ability of the SOX17 mutants to repress WNT signaling in EC, we used the well-characterized pBAR TCF/β-catenin activated luciferase reporter (also called TOPflash) [35]. Forced expression of SOX17 repressed β-catenin signaling measured by pBAR in SW480 and HCT-116 colorectal cancer (CRC) cell lines, consistent with reported data [22, 34] (Supplementary Figure 5). However, when the same plasmids were transfected into each of three EC cell lines, no repression was seen with any of the SOX17-WT or mutant constructs (Figure 4A). There was also no repression caused by transfection of SOX17-WT or mutant constructs in EC cell lines co-transfected with a mutant form of β-catenin with enhanced stability, indicating that the observed differences between EC and CRC cells were not due to differences in basal levels of β-catenin (Figure 4B).

To further explore the relationship between SOX17 and β-catenin in EEC, primary EECs harboring mutations in *SOX17* or *CTNNB1* (the gene that encodes B-catenin) were co-stained for both proteins. There was no evidence of enhanced or reduced staining in mutant samples, and we did not see evidence for tissue specific co-expression or exclusivity of SOX17 and β-catenin in any of the specimens examined (Supplementary Figure 6). These data may indicate that the cross-talk between SOX17 and β-catenin evident in developing tissues and other cancer types is not preserved in EC.

## DISCUSSION

The *SOX17* mutation pattern we observed in EECs is consistent with that of a tumor suppressor, in which critical regions are perturbed through single amino acid substitutions, and nonsense and frameshift mutations occur throughout the entire coding sequence. *SOX17* has been implicated as a tumor suppressor gene in various solid tumors [22, 30, 36, 37], and we argue that the mutations we detected occur because of cancer-specific selection rather than mutational noise because of the small size of the gene, the paucity of *SOX17* mutations in *POLE*-mutated tumors and the lack of strand-slippage mutations in MSI tumors. However, the absence of any cases displaying loss of heterozygosity or multiple *SOX17* mutations (i.e. no second hit mutations), and the functional differences between the missense and frameshift mutations indicate that the role of *SOX17* mutations in EEC is unlike classical tumor suppressor genes.

Zhang and colleagues recently used a small number of cases to determine that ECs with reduced SOX17 levels are significantly more often advanced stage and these patients had significantly reduced recurrence-free survival [23]. Our findings support the association between SOX17 expression with advanced EC stage and poor outcome (Supplementary Figure 1 and Supplementary Table 3). We also identified a significant association between *SOX17* mutation status and tumor grade (Supplementary Table 2). This association could in part be explained by the higher rate of *SOX17* mutation in MSI tumors that are known to be more frequently grade 2 tumors [38].

Our functional characterization efforts revealed that the hotspot missense mutations, p.Arg96Gly and p.Ser403Ile, did not affect SOX17-mediated transcriptional activity, but forced expression of SOX17-A96G and SOX17-S403I reduced the viability of transfected EC cell lines even further than transfection with SOX17-WT. This result was unexpected, as we hypothesized that these mutations would negatively affect SOX17 tumor suppressor activity and potentially allow cells to escape the reduced proliferation caused by SOX17. It remains to be seen if these mutations truly inhibit the proliferation of EC in human tumors, or whether this is an artifact of cell line models and *in vitro* culture system.

Interestingly, transfection of the HEC1A and Ishikawa EC cell lines with SOX17-WT did not affect luciferase driven by pBAR (Figure 4). This is in contrast to the SOX17-mediated repression of WNT signaling reported by other groups using the same pBAR reporter plasmid [23, 39, 40]. SOX17 does not directly interact with the reporter, but instead it is an indirect measurement of SOX17's modulation of TCF and β-catenin activity. The effects of SOX17 on pBAR activity has been measured by multiple investigators in multiple cell types and inconsistencies have been described [40]. We speculate that there is a fine balance between SOX17's activation and repression of pBAR that is influenced by multiple factors including: the induction of SOX17 by β-catenin [41], the GSK3β-independent downregulation of β-catenin by SOX17 [34, 42], the direct interaction of SOX17, TCF and β-catenin [33, 34], the similarity between TCF and SOX17 binding motifs [43], the possibility that SOX17-mediated repression of TCF promoter sites involves additional WNT-induced factors other than β-catenin [40], and the positive feedback loop mediated by TCF-4 sites within the β-catenin promoter [44].

Overall, our identification and characterization of both missense and frameshift mutations in *SOX17* expands upon TCGA's earlier discovery of *SOX17* missense mutations in EC [5]. Our data show that half of *SOX17* mutations are loss-of-function defects, many of which result in loss of transcriptional activity, which further highlights the importance of SOX17 in endometrial tumorigenesis. Gene-by-gene characterization of mutational targets in EC is critical for moving towards a comprehensive understanding of the biology of this tumor type. Our data link *SOX17*, a critical regulator of development that plays a role in proper formation of the uterus with uterine endometrial carcinoma and implicate *SOX17* as another player involved in EC pathobiology.

## MATERIALS AND METHODS

### Patient materials

Tumor samples from patients being treated for uterine cancer were collected by the Division of Gynecologic Oncology, Washington University Medical Center (approved protocols HSC 91-0507 and HSC 93-0828), from 1991-2010. MSI testing was previously performed [45, 46], using the 5 NCI consensus markers (BAT25, BAT26, D2S123, D5S346, and D17S250). *POLE* proofreading domain mutation screening was previously performed [25]. Surgical staging and tumor grade was assigned based on the basis of FIGO 2009 criteria [47, 48].

### Tumor sequencing

Both *SOX17* coding exons were sequenced in 539 EEC samples using a combination of next-generation sequencing (NGS) and Sanger methods. Primers and conditions are supplied in Supplementary Table 4. All variant calls (Sanger and NGS) were sequenced by Sanger methods in paired germline DNA to determine somatic origin. Targeted deep sequencing library preparation was performed using the TruSeq Custom Amplicon Kit v1.5 (Illumina, San Diego, CA), with amplicons targeting both *SOX17* exons and the 3’ UTR. Bar-coded and amplified specimens were multiplexed and sequencing was performed on an Illumina MiSeq® using 250 base paired end reads (MiSeq Reagent Kit v2). Variants were identified using Miseq Reporter^TM^ software version 2.5.1 with the GATK variant caller [49, 50].

Sanger sequencing was performed on all tumors for a GC-rich 832bp region of exon 2 that was poorly covered by the target panel (0 reads in most samples), and for all of exon 1 in the 43 tumors that were poorly covered for the exon 1 amplicons (<40X average read depth). Sanger sequencing was performed for an additional 12 tumors to validate the variants detected by targeted deep sequencing.

### Immunofluorescence microscopy

For cell line studies, cells were seeded in 6-well plates on coverslips, then transfected with wild-type (WT) or mutant SOX17 expression constructs using the ProFection® Mammalian Transfection System (Promega, Madison, WI). Forty-eight hours after transfection, cells were fixed using 3.7% formalin, permeablized with 0.1% triton-100, blocked with 4% normal goat serum, then stained with indicated primary antibody (and subsequent Alexa Fluor-488 secondary antibody) and DAPI. Primary tumor sections (4μm thick) were deparaffinized, then heated in 10mM sodium citrate buffer (pH 6.0) containing 0.05% Tween for 20 minutes in an Electric Pressure Cooker. Sections were blocked with 2% donkey serum and 1% BSA then incubated with indicated primary antibodies, followed by corresponding secondary antibodies and bisbenzimide H 33258 (Hoechst 33258, Sigma-Aldrich, St. Louis, MO). Photomicrographs were captured using a BZ-9000 microscope (Keyence, Osaka, Japan). Microscope settings were not changed between samples and SOX17 intensity was scored by blinded researchers as absent/low, moderate or high (Supplementary Figure 7).

### Viability assay

The indicated cell lines were seeded in opaque 96-well plates, then transfected with WT or mutant SOX17 expression constructs using Lipofectamine 2000 (Thermo Fisher, Waltham, MA). Viability was assessed every 24 hours using chemiluminescent TiterGlo assays (Promega, Madison, WI) from three biological replicates according to the manufacturer's instructions.

### Cell cycle analysis

AN3CA cells expressing EV, SOX17-WT, SOX17-A96G and SOX17-S403I fixed were in ethanol, treated with RNAse A and then suspended in propidium iodide. Fluorescence-activated cell sorting was performed using an LSR II cytometry (Beckman-Coulter, Brea, CA, USA), with 10,000 events recorded per condition. Cell cycle analysis was performed with the FlowJo software v7.6.3 (FlowJo LLC, Ashland, OR, USA) using the Watson model and no constraints.

### COBRA and bisulfite sequencing

Bisulfite conversion of primary tissue DNA was performed using EZ DNA Methylation-Gold Kit reagents (Zymo Research), Irvine, CA. The SOX17 promoter region was amplified as described [30]. Digestion was performed with either HhaI or TaqI, then DNA fragments were resolved on 10% polyacrylamide gels. PCR products were cloned using the PCR-2.1TOPO TA vector (Invitrogen, Carlsbad, CA) and sequenced using the M13 reverse primer.

### Luciferase reporter assay

Cells were seeded in 12-well plates and transfected using Lipofectamine 2000 (Thermo Fisher, Waltham, MA) with the indicated expression plasmids (200ng unless otherwise indicated), renilla luciferase control plasmid (50ng), and a firefly luciferase reporter (200ng unless otherwise indicated). Luciferase was measured via the Dual-Luciferase Reporter System (Promega, Madison, WI).

### Cell culture

293T cells and AN3CA cells were cultured in DMEM (Life Technologies, Carlsbad, CA), Hec-1a cells were cultured in McCoy's 5A media (Life Technologies, Carlsbad, CA), and Ishikawa cells were cultured in 1:1 F12:DMEM (Life Technologies, Carlsbad, CA). All cell lines were cultured with 10% FBS (Life Technologies, Carlsbad, CA). Cell lines used were confirmed to be mycoplasma negative using the MycoAlert Mycoplasma Detection Kit (Lonza, Basel, Switzerland). Cells were obtained from American Type Culture Collection.

### Western blotting

Cells were lysed with RIPA buffer, and lysates were subjected to sodium dodecyl sulfate- polyacrylamide gel electrophoresis followed by nitrocellulose membrane transfer. Membranes were blocked with non-fat dry milk, and probed with the indicated primary antibody, then HRP-conjugated secondary antibody.

### Antibodies and plasmids

The following antibodies were used: c-Myc (9E10) (sc-40, Santa Cruz Biotechnology, Santa Cruz, CA); SOX17 (AF1974, R&D systems, Minneapolis, MN); pan-cytokeratin (sc-81714, Santa Cruz Biotechnology, Santa Cruz, CA); β-catenin (zymed 13-8400, Thermo Fisher, Waltham, MA) vinculin (v4139, Sigma-Aldrich, St. Louis, MO); HRP-anti-mouse IgG (NA934V, GE Healthcare); Alexa Fluor-488 goat anti-mouse IgG (H+L) (A11029, Invitrogen, Carlsbad, CA), Alexa Fluor-488 donkey anti-goat IgG (H+L) (705-546-147, Jackson ImmunoResearch Laboratories, West Grove, PA); Alexa Fluor-594 donkey anti-mouse IgG (H+L) (715-586-150, Jackson ImmunoResearch Laboratories, West Grove, PA).

The coding sequencing of WT *SOX17* was PCR-amplified from human tumor cDNA synthesized using superscript III reverse transcriptase (Thermo Fisher, Waltham, MA), and cloned into the pCDH-CMV-MCS-EF1-GreenPuro plasmid (SystemsBiosciences Palo Alto, CA). Mutations were introduced using the QuikChange II XL Site-Directed Mutagenesis Kit (Agilent Technologies, Santa Clara, CA). A mutant β-catenin (S33A, S37A, T41A, S45A) expression construct, SOX17 compressed motif reporter [16] and pBAR TCF/β-catenin activated reporter [35] were obtained from addgene.

### Statistical analysis

P-values for survival were calculated by log-rank test. Significance for associations between *SOX17* mutation status and clincopathologic and demographic variables was calculated by Fisher's exact tests. Testing for associations with SOX17 protein expression was performed by dichotomizing variables as follows: low/absent expression and medium/high expression; *SOX17* mutant and *SOX17* wild-type; grade 1 and grade 2/3; stage I/II and stage III/IV, and using Fisher's exact tests for significance. For viability assays significance was determined for each time point by one-way ANOVA and Tukey's multiple comparison test. For luciferase assays shown in Figure 4, significance was determined by Tukey's multiple comparison test between all groups. For cell cycle analysis significance was determined by Tukey's multiple comparison test. Calculations were performed using Prism 5 (GraphPad Software, La Jolla, CA).

## SUPPLEMENTARY TABLES AND FIGURES









## Abbreviations

CRC: colorectal cancer
EC: endometrial cancer
EECs: endometrioid endometrial carcinomas
FIGO: Fédération Internationale de Gynécologie et d’Obstétrique
LOF: loss-of-function
MSI: microsatellite instability
NGS: next-generation sequencing
SCNA: somatic copy number alteration
TCGA: The Cancer Genome Atlas
WT: wild-type
