# Supplementary material for: Novel SOX17 frameshift mutations in endometrial cancer are functionally distinct from recurrent missense mutations
Source: Oncotarget. 2017 Aug 12;8(40):68758–68. doi: 10.18632/oncotarget.20213 (PMC5620294; doi:10.18632/oncotarget.20213)
Supplement: Supplementary file 1 [file oncotarget-08-68758-s001.pdf]

Novel *SOX17* frameshift mutations in endometrial cancer are functionally distinct from recurrent missense mutations

SUPPLEMENTARY MATERIALS

Supplementary Table 1: Mutations observed in 539 endometrioid endometrial carcinoma tumors.

See Supplementary File 1

Supplementary Table 2: Clincopathologic and demographic associations with SOX17 mutations in 539 endometrioid endometrial carcinomas.

See Supplementary File 2

Supplementary Table 3: Correlation between SOX17 protein levels with mutation, tumor stage and grade.

See Supplementary File 3

Supplementary Table 4: Primers and cycling conditions used for *SOX17* Sanger sequencing

Primers	Cycling	Composition
Asox17ex1_F2: TGGGTACGCTGTAGACCAGA	95°C for 3 min	
Asox17ex1_R2: CTACACACCCCTGGTTTTGG	34 cycles of 95° C for 1 minute, 60°C for 1 minute and 72°C for 1 minute 72° C for 5 minutes	AmpliTaq® Gold 10% DMSO
Sox17_Ex2_F: CGGTTGCGCAATTCAAAGTC	95°C for 3 min	
Sox17_Ex2_R: GATCAGGGACCTGTCACACG	36 cycles of 95° C for 1 minute, 58° C for 1 minute and 58°C for 1 minute 72° C for 5 minutes	AmpliTaq® Gold 10% DMSO
Sox17_MF:CAGGACCACCCCAACTACAA	95°C for min	
Sox17_Ex2_R: GATCAGGGACCTGTCACACG	34 cycles cycles of 95° C for 1 minute, 60° C for 1 minute and 72°C for 1 minute	AmpliTaq® Gold 10% DMSO
*Sox17_Ex2_Fseq: TGGCCATGGACGGCCTG	72°C for 5 minutes	

*Sox17_Ex2_Fseq is used as a nested sequencing primer.

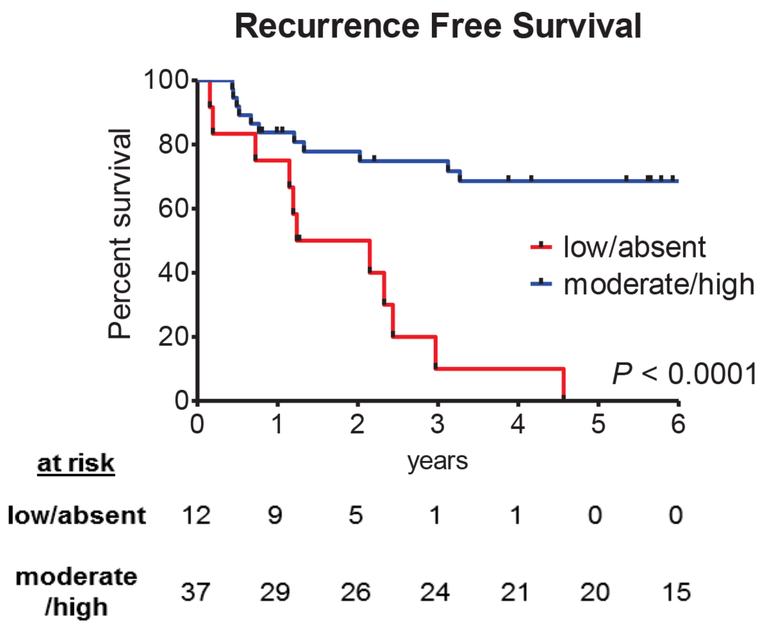

Supplementary Figure 1: SOX17 expression is associated with reduced recurrence-free survival. Fifty-one tumors were stained for SOX17 expression and scored as low, absent, moderate or high. Tumors with low/absent SOX17 had reduced recurrence-free survival compared to those with moderate/high expression. Two patients with progressive disease were excluded. *P*-value calculated by log-rank test.

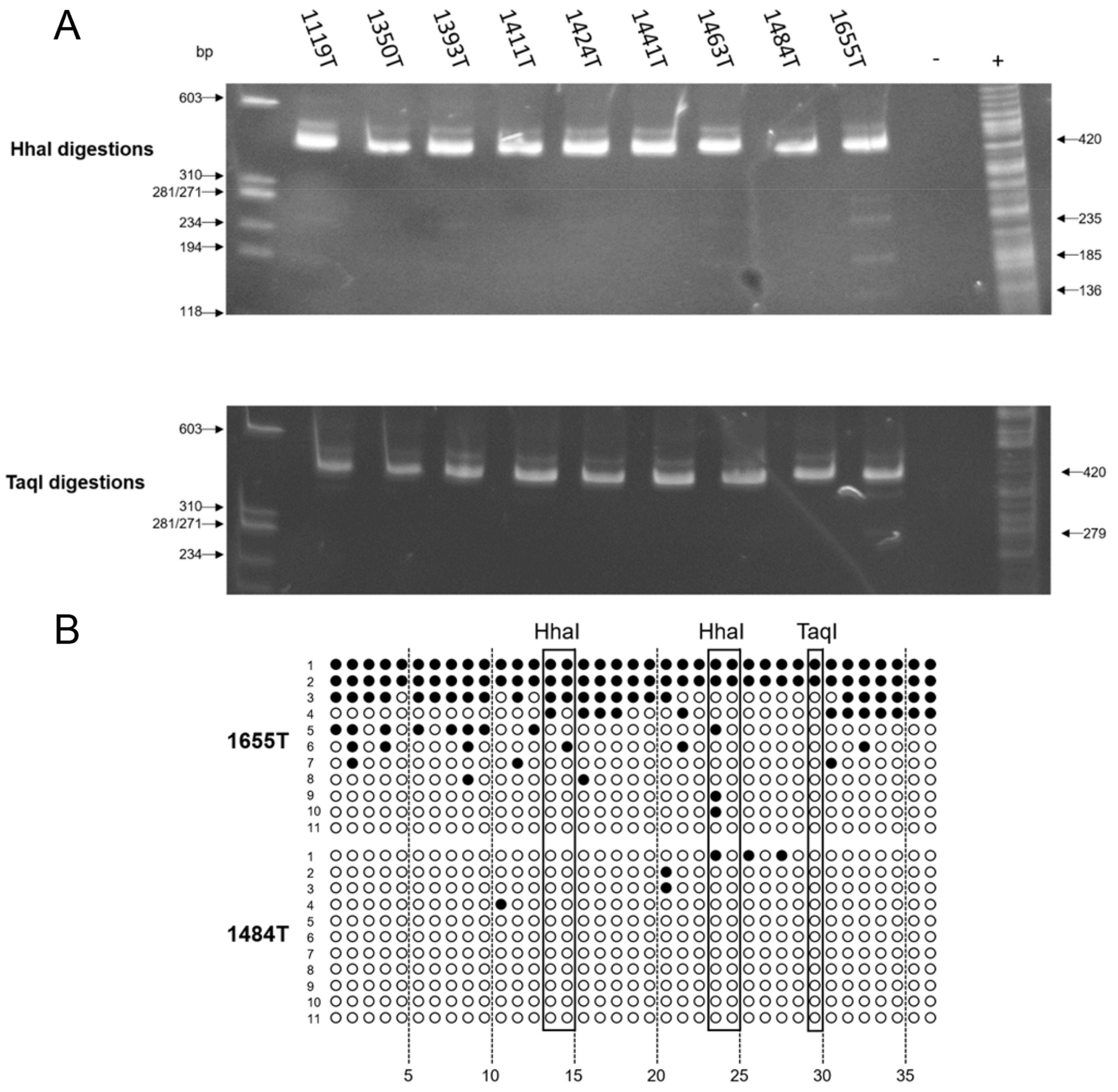

Supplementary Figure 2: The *SOX17* promoter region is infrequently methylated in EEC. (A) The indicated primary tumor DNAs were bisulfite converted then used to amplify the 420bp *SOX17* promoter region. PCR products were digested with either HhaI or TaqI. The sub-420bp sized bands present in the 1655T lane indicate the PCR product was digested, and therefore the CpG sites within the restriction sites were methylated. No-template PCR is included as negative control and digestion of λ DNA-HindIII is shown as positive control. **(B)** Eleven individual PCR amplicons from tumor 1655T and 1484T (negative control) were topo cloned and Sanger sequenced. Plot shows the conversion status of the 37 CpG sites that were evaluable in the *SOX17* promoter (black is not converted, and white is converted). HhaI and TaqI sites used for COBRA are indicated by black boxes.

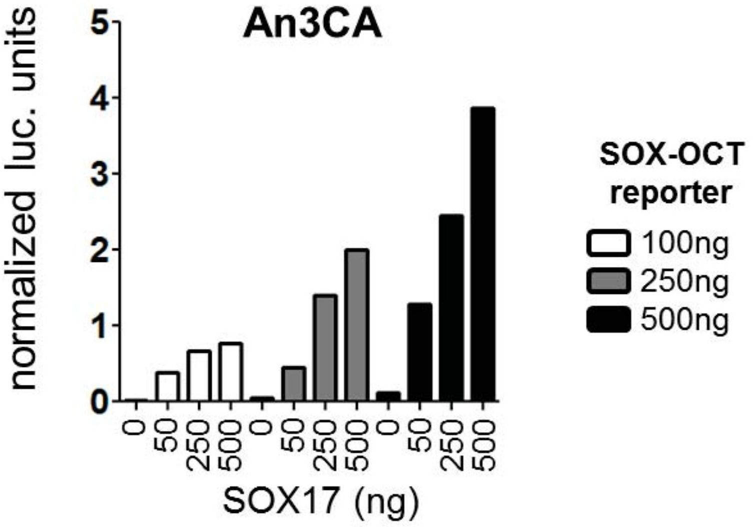

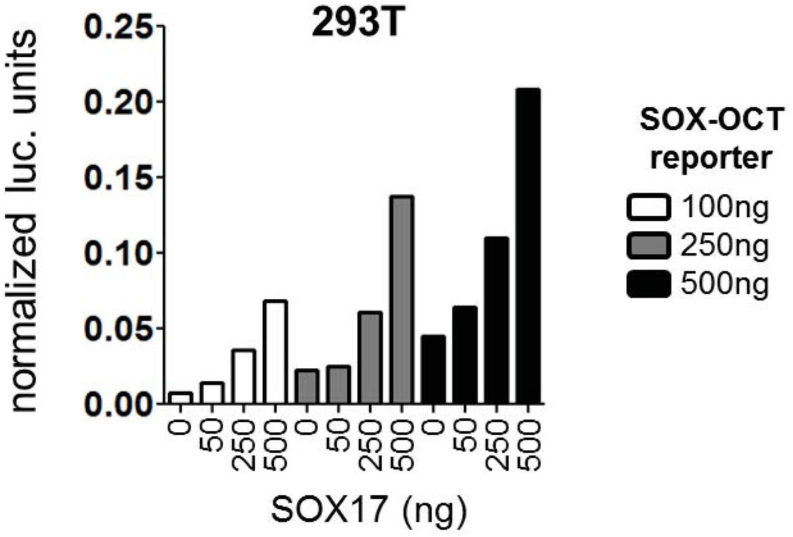

Supplementary Figure 3: The SOX-OCT compressed motif luciferase reporter is responsive to SOX17. An3CA (top) and 293T (bottom) cells were transfected with increasing amount amounts of the SOX-OCT compressed motif firefly luciferase reporter, increasing amounts of wild-type SOX17, and a control renilla luciferase plasmid. The reporter is responsive to SOX17, as indicated by increasing activity with increasing amount of transfected SOX17. Graphs show the firefly/renilla lucierase ratio (normalized luciferase units).

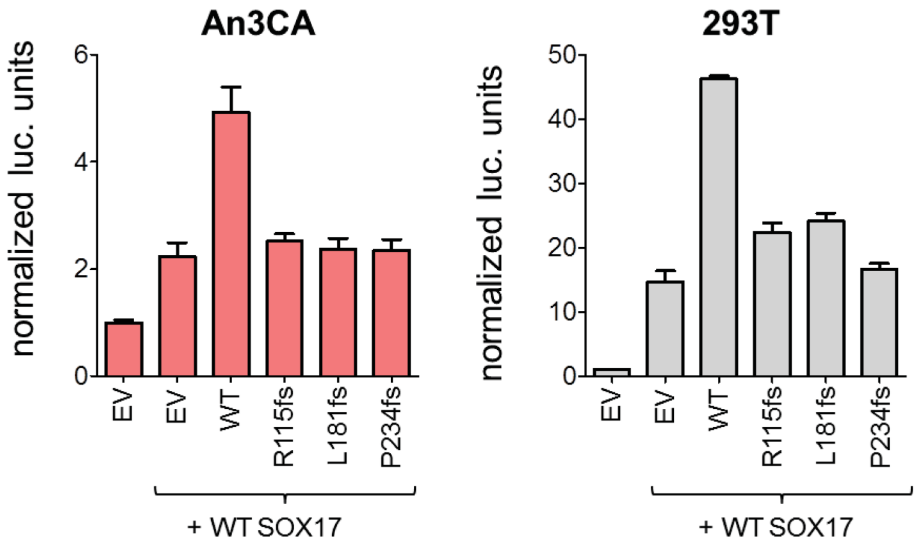

Supplementary Figure 4: SOX17 frameshift mutants do not act as dominant negatives. An3CA or 293T cells were co-transfected with the indicated SOX17 frameshift mutant constructs and equal amounts of the SOX17 wild-type construct, along with the compressed SOX-OCT luciferase reporter and control renilla plasmid. To control for total DNA the amount of empty vector (EV) was doubled when transfected without any SOX17 (EV alone bar). Graphs show the firefly/renilla luciferase ratio (normalized luciferase units).

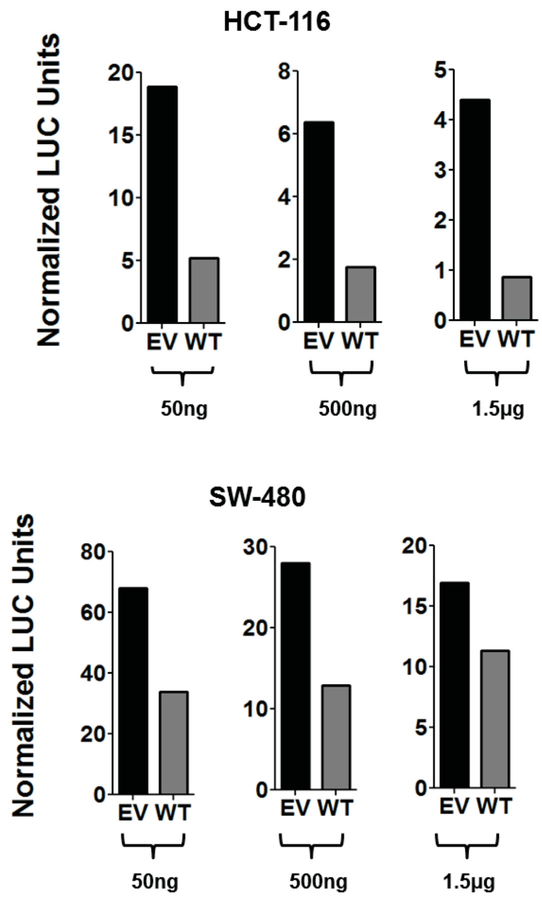

Supplementary Figure 5: Forced expression of SOX17 reduces TCF/β-catenin transcriptional activity in colorectal cancer cell lines. Increasing amounts of empty vector (EV) and wild-type (WT) SOX17 were co-transfected with a β-catenin activated firefly luciferase reporter (pBAR) and a control renilla luciferase plasmid into HCT-116 cells (top) and SW-480 cells (bottom). Firefly to renilla luciferase ratio is reported (normalized LUC units).

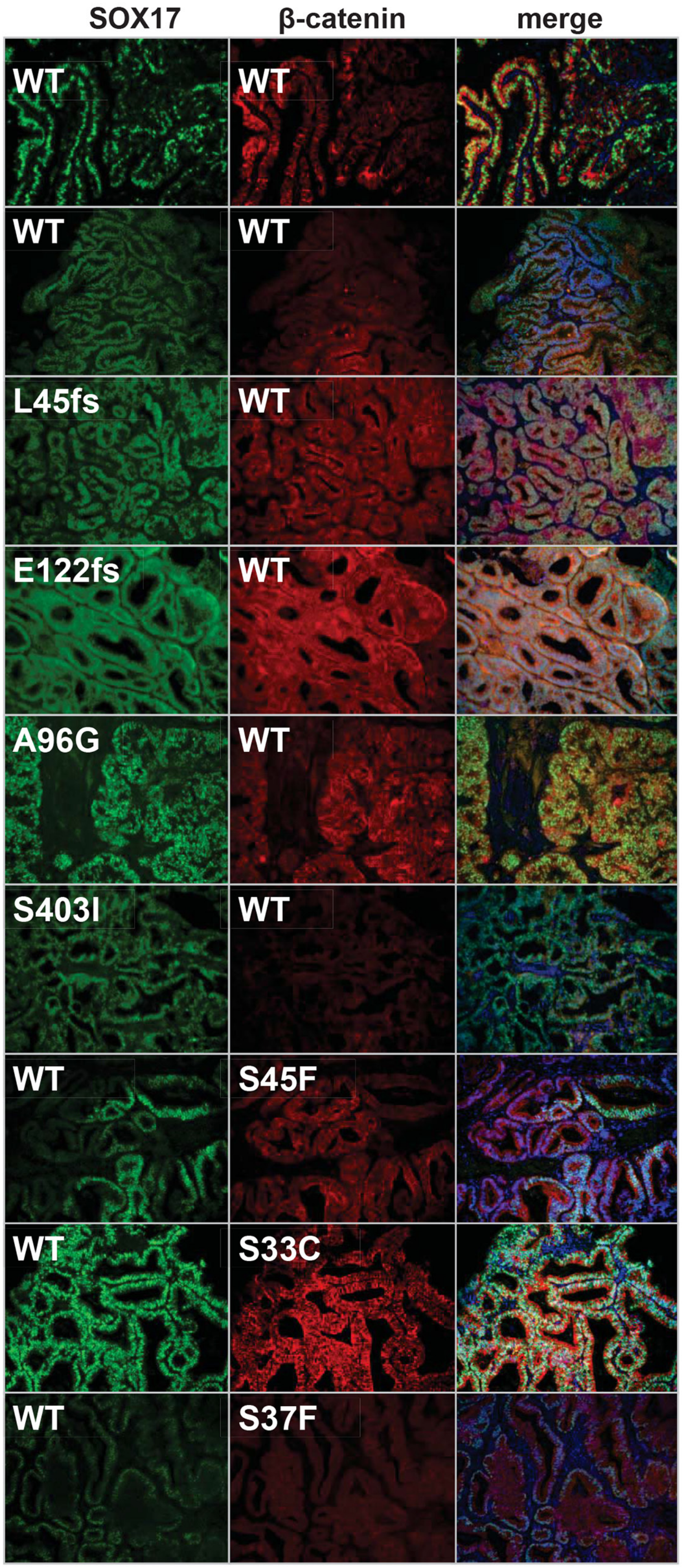

Supplementary Figure 6: SOX17 levels do not correlate with β-catenin expression or mutation status in EC. Immunofluorescence microscopy for SOX17 and β-catenin was performed for 15 tumors, 5 of which harbored *SOX17* mutations, 5 with *CTNNB1* mutations and 5 wild-type (WT). Sample images are shown for WT and mutant tumors. No clear relationships between expression patterns and mutation status were evident.

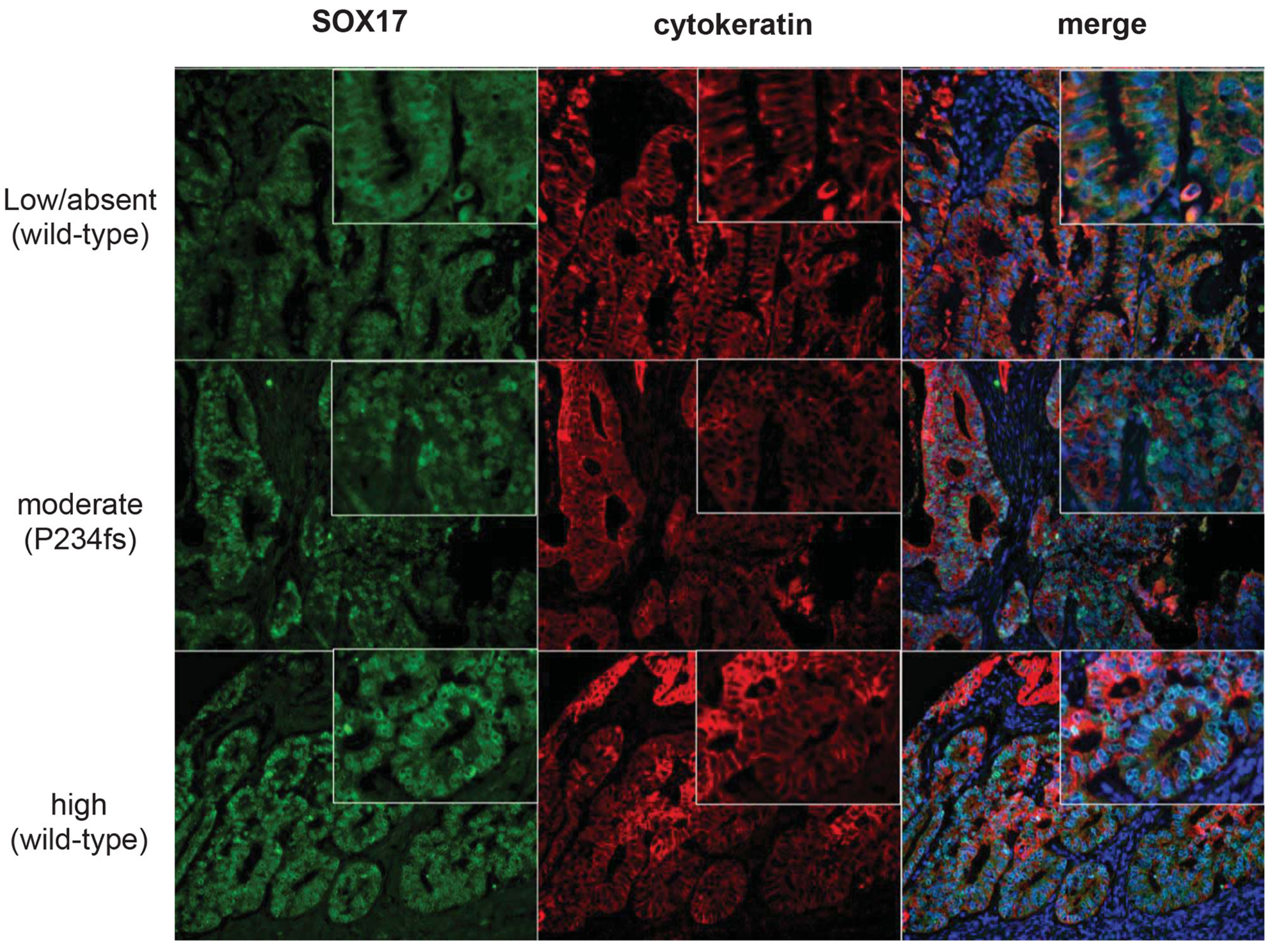

Supplementary Figure 7: Variable SOX17 expression in primary EECs. Representative images of EECs with low/absent, moderate and high SOX17 expression in cell nuclei. The levels of SOX17 protein did not correlate with *SOX17* mutation status. Cytokeratin counter-stain shows epithelial cells. See also Supplementary Table 3.
