# Supplementary material for: Novel SOX17 frameshift mutations in endometrial cancer are functionally distinct from recurrent missense mutations
Source: Oncotarget. 2017 Aug 12;8(40):68758–68. doi: 10.18632/oncotarget.20213 (PMC5620294; doi:10.18632/oncotarget.20213)
Supplement: Supplementary file 2 [file oncotarget-08-68758-s002.docx]

**Supplementary Table 1: Mutations observed in 539 endometrioid endometrial carcinoma tumors**

| **Mutation** | | **Cases** | | | |  |  | **Length of frameshift protein** |
| --- | --- | --- | --- | --- | --- | --- | --- | --- |
| **Protein** | **cDNA** | **total MSS MSI POLE** | | | | **Repeat** | **Condel** |  |
| p.C30fs*51 | c.80dup | **1** | 1 | . | . | . | . | 79 |
| p.K45fs*36 | c.131dup | **1** | 1 | . | . | . | . | 79 |
| p.A74T | c.220G>A | **1** | . | 1 | . | . | del. | . |
| p.A74V | c.221C>T | **1** | 1 | . | . | . | del. | . |
| p.W78fs*3 | c.230dup | **1** | . | 1 | . | . | . | 79 |
| p.K80* | c.237dup | **1** | 1 | . | . | . | . | . |
| p.A96G | c.287C>G | **8** | 4 | 4 | . | . | del. | . |
| p.L98V | c.292T>G | **1** | . | 1 | . | . | del. | . |
| p.K100N | c.300G>T | **1** | . | 1 | . | . | del. | . |
| p.G103D | c.308G>A | **1** | . | 1 | . | . | del. | . |
| p.E113* | c.337G>T | **1** | 1 | . | . | . | . | . |
| p.R115fs*47 | c.341dup | **1** | 1 | . | . | . | . | 160 |
| p.E122fs*39 | c.365_366delAG | **1** | . | 1 | . | . | . | 159 |
| p.H132R | c.395A>G | **1** | . | 1 | . | . | del. | . |
| p.K136fs*11 | c.403_404dup | **1** | . | 1 | . | . | . | 145 |
| p.W135fs*27 | c.403dup | **1** | . | 1 | . | . | . | 160 |
| p.K136E | c.406A>G | **2** | . | 2 | . | . | del. | . |
| p.K136N | c.408G>C | **1** | 1 | . | . | . | del. | . |
| p.K149fs*13 | c.443dup | **1** | . | 1 | . | . | . | 160 |
| p.L156fs*6 | c.464dup | **1** | 1 | 1 | . | . | . | 160 |
|  | c.480_495delTGA |  |  |  |  |  |  |  |
| p.E161fs*221 | GCCGCAGGCGG |  |  |  |  |  |  | 380 |
|  | CC | **1** | . | 1 | . | . | . |  |
| p.Q163fs*217 | c.487_508del | **1** | . | 1 | . | . | . | 378 |
| p.L167fs*213 | c.499_520del | **3** | 2 | 1 | . | . | . | 378 |
| p.L181fs*184 | c.539dup | **1** | 1 | . | . | . | . | 363 |
| p.Q182H | c.546G>T | **1** | . | . | 1 | . | neut. | . |
| p.E185fs*180 | c.548dup | **1** | . | 1 | . | . | . | 363 |
| p.Q186fs*179 | c.556dup | **1** | . | 1 | . | . | . | 363 |
| p.P189fs*199 | c.564_565insTT | **1** | . | 1 | . | . | . | 386 |
| p.L209fs*155 | c.622_623del | **1** | 1 | . | . | . | . | 362 |
| p.Q324_H325del | c.972_977del | **2** | 1 | 1 | . | CACCAGx4 | . | . |
| p.P234fs*153 | c.701delC | **1** | . | 1 | . | . | . | 385 |
| p.Q255H | c.765G>T | **1** | . | 1 | . | . | neut. | . |
| p.P263fs*124 | c.788delC | **2** | . | 2 | . | Cx6 | . | 385 |
| p.E264fs*101 | c.788dup | **2** | . | 2 | . | Cx6 | . | 363 |
| p.R273fs*114 | c.817delC | **1** | . | 1 | . | Cx4 | . | 385 |
| p.I283fs*81 | c.846_847del | **1** | . | 1 | . | . | . | 362 |
| p.His326del_insQH QPP | c.977_978insGCA CCAGCCCCC | **1** | . | 1 | . | CACCAGx4 | . | . |
| p.P328fs*59 | c.983delC | **3** | . | 3 | . | Cx5 | . | 385 |
| p.Q350fs*14 | c.1044_1045del | **1** | . | 1 | . | . | . | 362 |
| p.Q380fs*4 | c.1138dup | **1** | 1 | . | . | . | . | 382 |
| p.N387fs*67 | c.1160delA | **1** | 1 | . | . | . | . | 452 |
| p.S403I | c.1208G>T | **6** | 6 | . | . | . | del. | . |

Mutations listed correspond to transcript NM_022454.3. MSI, microsatellite instable; MSS, microsatellite stable; del, deleterious; neut, neutral
