# Supplementary material for: Novel SOX17 frameshift mutations in endometrial cancer are functionally distinct from recurrent missense mutations
Source: Oncotarget. 2017 Aug 12;8(40):68758–68. doi: 10.18632/oncotarget.20213 (PMC5620294; doi:10.18632/oncotarget.20213)
Supplement: Supplementary file 3 [file oncotarget-08-68758-s003.docx]

**Supplementary Table 2: Clincopathologic and demographic associations with SOX17 mutations in 539 endometrioid endometrial carcinomas**

**no. of Patients (%)**

**Parameter**

**wild-type (n**

**= 477)**

***SOX17* mutated**

**(n = 62)**

*P*

MSI/*POLE* Status

Grade

1

2

3

246 (52)

156 (33)

75 (16)

23 (37)

31 (50)

8 (13)

0.026

| MSS (POLE WT) | 272 (57) | 25 (40) | 0.005 |
| --- | --- | --- | --- |
| MSI (POLE WT) | 178 (37) | 36 (58) |  |
| *POLE* mutated | 27 (6) | 1 (2) |  |

MSI, microsatellite unstable; MSS, microsatellite stable; LVSI, lymphovascular space invasion

| Age,y  <60 | 202 (42) | 18 (29) | ns |
| --- | --- | --- | --- |
| >60 | 275 (58) | 44 (71) |  |
| LVSI  Absent | 309 (66) | 39 (64) | ns |
| Present | 158 (34) | 22 (36) |  |
| Stage  Early: I & II | 388 (82) | 53 (85) | ns |
| Advanced: III & IV | 88 (18) | 9 (15) |  |
| BMI, kg/m2  <30 | 146 (35) | 17 (30) | ns |
| 30-40 | 157 (37) | 23 (41) |  |
| >40 | 117 (28) | 16 (29) |  |
| Race  White | 422 (88) | 53 (86) | ns |
| Non-white | 55 (12) | 9 (14) |  |
