# Supplementary material for: Novel SOX17 frameshift mutations in endometrial cancer are functionally distinct from recurrent missense mutations
Source: Oncotarget. 2017 Aug 12;8(40):68758–68. doi: 10.18632/oncotarget.20213 (PMC5620294; doi:10.18632/oncotarget.20213)
Supplement: Supplementary file 4 [file oncotarget-08-68758-s004.docx]

**Supplementary Table 3: Correlation between SOX17 protein levels with mutation, tumor stage and grade**

| **sample number** | ***SOX17***  **expression** | ***SOX17***  **mutation** | **tumor grade** | **tumor stage** |
| --- | --- | --- | --- | --- |
| 1282 | low/absent | wt | 2 | IIIA |
| 1284 | low/absent | wt | 1 | IA |
| 1352 | low/absent | wt | 3 | IIIC2 |
| 1411 | low/absent | p.Trp135fs*27 | 2 | IIIC1 |
| 1419 | low/absent | wt | 3 | IIIC1 |
| 1424 | low/absent | wt | 2 | IA |
| 1441 | low/absent | wt | 3 | IIIC1 |
| 1474 | low/absent | p.Ser403Ile | 3 | IIIC2 |
| 1484 | low/absent | wt | 3 | IIIC2 |
| 1501 | low/absent | wt | 3 | IA |
| 1655 | low/absent | wt | 2 | IIIC1 |
| 1707 | low/absent | wt | 2 | IIIC2 |
| 1094 | medium | wt | 1 | IA |
| 1194 | medium | wt | 3 | IIIC2 |
| 1193 | medium | wt | 1 | II |
| 1205 | medium | wt | 1 | IB |
| 1209 | medium | wt | 2 | IA |
| 1221 | medium | wt | 1 | IB |
| 1226 | medium | wt | 2 | IVB |
| 1236 | medium | wt | 3 | IIIA |
| 1267 | medium | p.Pro328fs*59 | 2 | IB |
| 1289 | medium | wt | 3 | IB |
| 1316 | medium | wt | 1 | IIIC1 |
| 1391 | medium | wt | 2 | IIIC2 |
| 1393 | medium | wt | 2 | IIIC2 |
| 1482 | medium | p.Pro234fs*153 | 2 | IVA |
| 1495 | medium | wt | 1 | IA |
| 1601 | medium | wt | 2 | IA |
| 1611 | medium | wt | 1 | IB |
| 1632 | medium | wt | 2 | IA |
| 1644 | medium | wt | 3 | IA |
| 1650 | medium | wt | 1 | IA |
| 1463 | medium | wt | 1 | IIIC1 |
| 1464 | medium | wt | 3 | IB |
| 1471 | medium | wt | 1 | IA |
| 1513 | medium | p.Ser403Ile | 1 | IB |
| 1534 | medium | p.Ala96Gly | 2 | IA |
| 1570 | medium | p.Ser403Ile | 2 | IVB |
| 1928 | medium | p.Lys45fs*36 | 2 | IA |
| 2526 | medium | p.Glu122fs*39 | 2 | IA |
| 1119 | high | wt | 3 | IVB |
| 1184 | high | wt | 1 | IIIC1 |
| 1350 | high | wt | 3 | IIIC2 |
| 1359 | high | p.Ala96Gly | 2 | IA |

| 1370 | high | wt | 3 | IB |
| --- | --- | --- | --- | --- |
| 1112 | high | p.Pro263fs*124 | 2 | IB |
| 1487 | high | wt | 1 | IA |
| 1545 | high | p.Ala96Gly | 2 | IB |
| 1572 | high | p.Leu167fs*213 | 1 | IA |
| 1873 | high | wt | 1 | IA |
| 1957 | high | p.Leu156fs*6 | 1 | IA |
|  | *P*-values: | 0.471 | 0.042 | 0.016 |

Protein levels were measured by immunofluresence microscopy

of primary endometrioid endometrial cancers stained with anti- SOX17. *P*-values calculated by two-sided Fisher's exact test with samples grouped as follows: low/absent expression compared to medium or high expression; mutant compared to

wild-type (wt); grade 1 compared to grade 2 and 3; stage I and II compared to stage III and IV.
